# Point-of-Care Ultrasonography in Internal Medicine: Limitations and Pitfalls for Novice Users

**DOI:** 10.7759/cureus.43655

**Published:** 2023-08-17

**Authors:** Mohammed G Elhassan, Sarbjot Grewal, Negin Nezarat

**Affiliations:** 1 Internal Medicine, Saint Agnes Medical Center, Fresno, USA; 2 Internal Medicine, Saint Agnes Medical Center, Fresno , USA

**Keywords:** medical school training, postgraduate training, medical education, novice learners, internal medicine (general medicine), point-of-care ultrasound (pocus)

## Abstract

Point-of-care ultrasound (POCUS) is increasingly being adopted in the field of internal medicine, leading to the development of POCUS curricula in undergraduate and postgraduate medical education programs. Prominent internal medicine societies and organizations worldwide recognize the expanding utilization of POCUS by internal medicine physicians, emphasizing the need for practitioners to be aware of both its benefits and limitations. Despite the growing enthusiasm for POCUS, clinicians, particularly those with limited clinical experience, must be cautious regarding its inherent limitations and the potential impact on their clinical practice. This review aims to outline the limitations and potential drawbacks of POCUS for medical students, residents, and internists who wish to stay abreast of the escalating use of POCUS in internal medicine and have a desire, or have already commenced, to incorporate POCUS into their practice. Additionally, it provides recommendations for enhancing POCUS proficiency to mitigate these limitations.

## Introduction and background

What is point-of-care ultrasound?

Point-of-care ultrasound (POCUS) entails employing ultrasound techniques at the patient's bedside to address precise clinical inquiries that arise during encounters with patients. This approach diverges from conventional radiological ultrasound examinations commonly ordered by medical providers, executed by ultrasound technicians, and analyzed by radiologists (also known as consultative ultrasound). Consequently, POCUS can be regarded as an augmentation of physical examination and bedside evaluation [1-5].

POCUS and internal medicine

It is not surprising that POCUS initially grew into a medical practice along the lines of the two specialties that need quick bedside diagnostic skills: emergency medicine and critical care units. Its utility has now expanded beyond these two specialties, encompassing various medical training such as anesthesia, obstetrics and gynecology, nephrology, and rheumatology [6-10]. Recently, there has been an increased interest and enthusiasm for POCUS in general internal medicine, both in inpatient and outpatient settings [11-13]. This surge in enthusiasm can be attributed to the development of compact and affordable pocket-sized ultrasound devices, which are replacing the large and cumbersome cart-based ultrasound machines when it comes to personal use. Medical students, internal medicine residents, and faculty internists have demonstrated keen interest in POCUS and acknowledge its practical value [14-19]. Consequently, many residency programs are incorporating formal POCUS training into their curriculum. According to a survey conducted among internal medicine program directors, 35% of programs offer formal POCUS education to all residents through a structured curriculum, while 28% provide it to some residents mainly through elective rotations [20]. The most commonly taught diagnostic POCUS applications, as per that same report, include cardiac, lung, volume assessment, abdominal free fluid, pleural, bladder, lower extremity deep venous thrombosis (DVT), kidney, musculoskeletal, and thyroid ultrasound, in that order. This trend is also noticeable in undergraduate medical education [21], with a recent survey revealing that 57% of the surveyed medical schools have implemented a formal POCUS curriculum [22].

Potential benefits that POCUS can add to internal medicine practice

The use of POCUS has emerged as a prevailing practice and, in some cases, the standard of care in various bedside medical procedures, including arterial line insertion, central venous line insertion, thoracentesis, and paracentesis [23]. In addition to its procedural applications, clinical employment of POCUS has exhibited promising outcomes for healthcare professionals operating at the patient's bedside. A systematic review encompassing six studies demonstrated that POCUS facilitated alterations in the primary diagnosis in 18% of cases and contributed to the identification of relevant diagnoses in 24% of cases. Furthermore, three studies reported a reduction in the length of hospital stay [24]. Notably, a limited-scale prospective study involving 100 patients found a statistically significant association between the utilization of POCUS by the hospital's medical emergency team (also referred to as the rapid response team) and a higher frequency of accurate diagnoses when compared to the assessment of acutely ill patients without POCUS [25]. Numerous internal medicine societies and organizations across the United States, Canada, and Europe have acknowledged the expanding adoption of POCUS by internal medicine physicians, recognizing its extensive clinical applicability in routine practice. These entities actively promote awareness among physicians regarding both the advantages and limitations associated with POCUS implementation [12,20,22,26,27].

Should novice POCUS users be concerned about bringing it to their practice?

In spite of the growing enthusiasm surrounding the development and proliferation of POCUS utilization, it is imperative for clinicians, particularly novices, to exercise caution regarding the limitations of POCUS and its potential impact on their practice. Scant research exists pertaining to the integration of POCUS within the realm of daily internal medicine practice and its consequent influence on clinical outcomes. One plausible explanation is the historical dearth of POCUS curriculum available to internal medicine physicians and residents, in contrast to their counterparts in emergency medicine and critical care. The objective of this review is to elucidate the potential pitfalls and limitations of POCUS for medical students, residents, and internists who aspire to stay abreast of the escalating employment of POCUS in internal medicine, and are either interested in or have already implemented POCUS in their clinical practice.

## Review

General concerns and pitfalls related to POCUS use for novice users

POCUS Use and Associated Patient-Related Outcomes

The data on whether POCUS (outside of its use for procedures) improves patient-related outcomes is limited. One small randomized study in the ED by Atkinson et al. found that early use of POCUS in the ED for managing patients with undifferentiated hypotension in addition to standard care vs. standard care without POCUS did not show improvement in survival, inotropes or intravenous fluid use, ICU stay, or total length of stay [28]. This 2018 study found similar results to another earlier ED study published in 2006 by Melniker et al., where the use of POCUS also did not result in survival benefit in trauma patients, despite its benefit in shortening time to operative care and improving resource utilization [29]. In fact, one retrospective study, also in ED, raised concern that the use of POCUS prior to an intervention (fluid bolus or use of inotropic medication) was associated with an adjusted odds ratio for death of 1.41 compared to patients who were managed without POCUS [30]. The exact reasons behind these findings are not well known, but these studies were relatively small and, in the case of the Atkinson et al. study, had exclusion criteria that could have potentially excluded patients who are thought to benefit from POCUS the most (e.g., patients with ruptured abdominal aortic aneurysms). This study supported the notion that POCUS should probably be used to answer particular questions at the bedside rather than as a broad protocol with multiple exams on the same patient. Conducting multiple POCUS exams can take some time, and this, theoretically, can potentially delay resuscitation in an ED.

Stakeholders in one academic center (hospital leaders, hospitalists, and subspecialists) mentioned in a survey that clinical impact, efficiency, and time are the main determinants for the adoption of POCUS [31]. In that regard, if POCUS is not found to affect key clinical outcomes (e.g., mortality and readmission rate), then stakeholders might not think of POCUS training as a priority. The American College of Physicians, in their statement regarding the use of POCUS in the ED and medical wards for patients with dyspnea, found that evidence is very uncertain (insufficient) on mortality, time to diagnosis, and time to treatment. They also mentioned that POCUS "probably does not reduce the length of hospital stay," but they suggest that it "probably increases the proportion of correct diagnoses from 59% to 91%" [22]. More studies are needed to delineate the exact effect of POCUS use by internists on patients’ mortality and other important outcomes.

POCUS and Concerns Regarding Quality Measures

Quality measures are meant to help providers improve their skills by creating standards set by experts that novice learners need to match. So far, few medical societies have created initiatives to try and standardize the use of POCUS in different specialties, including internal medicine, reflecting the relative novelty of this skill [32]. Saving images and having them reviewed by experts is a major part of this process, and this requires human and technical resources that not all programs can afford. The optimum number of images an internist needs to obtain and interpret to acquire mastery of each POCUS skill is not well known yet. This is particularly important in ultrasound because it is operator-dependent, and hence novice users are encouraged to perform POCUS exams under appropriate supervision to continuously refine their skills, despite the ambiguity about what is considered to be a competency level to justify offering 'POCUS certification' [33]. 

POCUS and Concerns Regarding Training and Curriculum Development

The Accreditation Council for Graduate Medical Education (ACGME) uses entrustable professional activities (EPA) in six core competencies to assess when residents are ready for independent practice. No such EPAs exist for POCUS training during internal medicine residency training yet, but the potential for that has been demonstrated in one program [34]. In one survey, faculty internists in Canada and the US found that the top three barriers to learning POCUS are the need for more training, the lack of handheld devices, and the need for direct supervision. They cited the lack of handheld devices as a potential barrier, even though they seem to have machines available in their institution [19]. Some experts recommend that novice learners start with large cart-based devices because they are more likely to have better resolution than hand-held devices. In one review, that approach was particularly suggested for POCUS examination of solid abdominal organ pathologies [35]. The availability of faculty trained in POCUS is important for any program that wants to adopt a POCUS curriculum, but this can be a challenge for some hospitals since POCUS is a relatively new skill for internal medicine physicians and many did not have formal training during or after residency. In a randomized trial in one internal medicine program, the availability of hand-held devices without faculty supervision did not increase the usage of POCUS by trainees or their image acquisition skills [36]. One potential idea to implement in a POCUS curriculum is to have a software platform where residents can save images for faculty to review and provide constructive feedback when available, in addition to the traditional one-to-one feedback during direct clinical skills observation. This longitudinal program can potentially improve residents’ POCUS knowledge assessments [37-40]. 

POCUS and Concerns Regarding Physical Examination Skills

There has been some concern regarding the decline in physicians' physical examination skills, especially among young physicians and physicians-in-training [41]. Whether the growing use of ultrasound will deter trainees from honing their physical examination skills is not known [42]. One survey showed that internists are generally not worried about that [14]. The notion that POCUS is considered by many to be an extension of physical examination, as mentioned before, should actually make novice POCUS learners perform accurate physical examination first in order to get the most out of their POCUS exam. The Society of Bedside Medicine, for example, has a fellowship program that focuses on helping its members integrate bedside skills, which include history taking, physical examination, and POCUS [43]. Many undergraduate and postgraduate programs also have similar curriculum for their trainees, and the results of such programs have been encouraging [44]. 

POCUS and Concerns Regarding Medico-Legal Liability

Another concern is whether physicians’ use of POCUS can lead to lawsuit generation due to adverse effects as a result of such use. One recent retrospective review showed that POCUS use by internal medicine, family medicine, pediatrics, or critical care physicians did not lead to lawsuits or legal consequences [45]. In fact, another review revealed that very few lawsuit cases cited not using POCUS at all or not using it in a timely fashion as a potential cause of legal consequences [46]. Also, as more ultrasound images will be stored for review and billing purposes, one question arises regarding 'incidental findings' during exams done for particular specific indications that were missed and/or not addressed by the examiner in a timely manner. At the present time, probably very few studies (if any) have reported or examined actual patient harm as an outcome from using POCUS, but it is not clear whether this will change in the future as POCUS use seems to be expanding and growing.

Common system-based pitfalls related to POCUS use for novice users

With each POCUS examination come some pitfalls related to image acquisition and/or interpretation. It is important that novice learners pay attention to these pitfalls while practicing and learn how to avoid them since their findings can potentially affect the decision-making process. In addition to cognitive POCUS knowledge, most pitfalls can be learned and avoided with: (a) repetition and doing more exams under supervision; (b) looking at the target structure from more than one window or view; and (c) making sure to interpret the findings in the light of the clinical scenario and pre-test probability of the diagnosis the examiner is looking for. Here, we highlight common pitfalls that novice learners in internal medicine might face during their practice. 

Pitfalls Associated With POCUS Examination of the Heart

Evaluation of left ventricular (LV) contractility is a common application of POCUS. Examiners need to have insight into pitfalls associated with acquisition and quality of the image because estimating LV contractility in POCUS is usually done with visual inspection using three concepts: anterior mitral valve septal movement during systole; thickening of ventricular myocardium during systole; and symmetrical movement of LV walls towards the center of the LV cavity during systole (myocardium excursion) [47]. Recognizing LV systolic function as normal, mildly-to-moderately reduced, or severely reduced by internal medicine physicians was found to correlate with ejection fraction estimation reported in formal echocardiography [48]. For that purpose, quality of image plays an important role, and POCUS users need to re-evaluate the machine setting (e.g., gain and depth) and patient position (e.g., left lateral decubitus position for apical view) to improve image quality as much as possible. Novice users might find images obtained with cart-based machines to have better resolution than those obtained with hand-held devices, as stated earlier. In the left parasternal short axis view, using levels other than the papillary muscle level can under or overestimate LV contractility. Assessing LV contractility using only mitral valve early (E) point septal separation in patients with valvular disease (especially mitral stenosis or aortic regurgitation) may give an inaccurate eyeball estimation of LV contractility. Studies examining this modality usually exclude patients with valvular disease [49,50]. Therefore, it is important to use the three visual methods in different views before coming to a conclusion about gross cardiac systolic function. One of the other pitfalls, especially when the study is done in an emergency situation, is the LV foreshortening, where the apex in the apical view appears rounded instead of the normal pear shape because the ultrasound waves do not cut through the apex but rather a little anteriorly or posteriorly [51]. This might lead to an overestimation of LV contractility and can be overcome by slow probe positioning at the same window.

During the left parasternal long axis cardiac view examination, make sure not to confuse the apical epicardial fat (which looks more heterogenous and moves with heart beats) for pericardial effusion (which is anechoic, does not move with heart beats, and can be seen in other cardiac views as well) (Video 1).

**Video 1:** Left parasternal long axis cadiac view showing a large epicardial fat pad (anterior to the right ventricular wall) Pericardial fat pad in this cardiac view can be mistaken for pericardial effusion. Pericardial fat is mostly seen in the long axis view, has a heterogenous appearance, and moves with contractions. Pericardial effusion appears anechoic and can be seen in more than one cardiac view.

Care also needs to be exercised not to confuse pericardial effusion with left-sided pleural effusion during the long-axis left parasternal cardiac exam. The relationship of the fluid to the descending aorta can be useful in that regard (Video 2).

**Video 2:** Left parasternal long axis cardiac view showing both pericardial effusion and left pleural effusion Note the relationship of pericardial effusion and left pleural effusion to the descending aorta (pericardial effusion runs anterior to it and the pericardial effusion runs posteriorly).

Findings of right ventricular (RV) strain with POCUS can be crucial in patients who present with acute chest pain or shortness of breath. When these patients are hemodynamically unstable, one important differential diagnosis associated with high mortality if untreated is acute pulmonary embolism (PE). While these findings can make a difference in management (treating with thrombolytics and/or thrombectomy first rather than anticoagulation), they are not specific to acute PE [52] (e.g., seen in any underlying cause of chronic pulmonary hypertension and RV infarction) and need to be interpreted in the appropriate clinical context (Videos 3-4).

**Video 3:** The D-shaped right ventricle in a patient presenting with acute chest pain and shortness of breath is found to have a PE PE: Pulmonary embolism

**Video 4:** The D-shaped right ventricle in another patient presenting with shortness of breath due to pulmonary hypertension

Identifying signs of large PE using POCUS requires some experience, and obtaining good-resolution images is important to making appropriate management decisions. In one survey, internal medicine residents’ confidence to identify these signs was among the lowest compared to other POCUS skills such as determining inferior vena cava (IVC) diameter and collapsibility and identifying pleural effusion [18]. 

Pitfalls Associated With POCUS Examination of the IVC

Probably the most common pitfall for novice learners is mistaking the aorta for the IVC. The aorta usually runs more posteriorly and has thicker walls, so pulsations can be appreciated. The IVC can be obtained from the aorta window by tilting (also known as fanning) the probe slowly towards the patient's right side (Video 5).

**Video 5:** The aorta seen first in this longitudinal subcostal cardiac view can be misinterpreted as the IVC The first larger and pulsating vessel (the aorta) can be mistaken for the smaller, more anterior vessel (the IVC), and can lead to misinterpretation of the exam as showing a large IVC. IVC: Inferior vena cava

When examining the maximum diameter of the IVC in the longitudinal view, it is important to avoid the 'cylinder effect', which is examining the IVC in an off-axis view, not the maximum diameter, and this can underestimate diameter and collapsibility and wrongly suggest hypovolemia. This can be easily avoided by slowly fanning the probe right and left over the full diameter of the IVC and measuring the maximum diameter seen. Although the correlation between IVC diameter and collapsibility with volume status is fair, other conditions can affect IVC diameter and distensibility, leading to variable and false results, especially in non-ventilated patients (e.g., large PE, pericardial tamponade, tricuspid valve disease, and positive pressure ventilation) [53]. 

*Pitfalls Associated With POCUS Examinations of the Lung*s

Novice POCUS users need to get acquainted with ultrasound lung artifacts seen in healthy and diseased lungs [54]. These artifacts are created because the lung is an air-filled organ, and air scatters ultrasound beams. Lung POCUS, in a way, is similar to the stethoscope: the probe looks at the pathology underneath and not the rest of the lung. For example, the presence of pleural sliding rules out pneumothorax only in the part of the lung being examined. Therefore, looking at multiple lung windows during the POCUS examination is important to minimize missing important findings underneath unexamined lung parts. Also, absent pleural sliding does not always indicate pneumothorax, and other pathologies need to be considered based on clinical scenarios (Video 6).

**Video 6:** Lung exam in the upper anterior zones, showing absent pleural sliding on the right side and normal sliding on the left side This lung exam in the upper anterior zones shows absent pleural sliding on the right side (with the M-mode showing the barcode sign) and normal sliding on the left side (with the M-mode showing a normal sand-on-the-beach appearance). This patient has a history of right decortication.

The finding of 'lung point' is said to be virtually diagnostic of pneumothorax, but care should be exercised not to confuse this useful sign with what looks like lung point but is not (e.g., the physiologic lung point near the mediastinal pleura, at the lung contusion site, over lung blebs, and others) [55-57]. Other findings, like absent B-lines and the barcode sign in M-mode, can support the presence of a true lung point, emphasizing the fact that it is always a good practice to confirm ultrasound findings with more than one view and mode and to look for associated signs as well [55]. Although cardiogenic pulmonary edema is the most common cause of bilateral B-lines, this finding has a broad differential diagnosis (including coronavirus disease 2019 and non-cardiogenic pulmonary edema), and it should always be interpreted in the appropriate clinical context. Careful examination of the number, pattern and shape of B-lines, the shape and contour of the pleural line, and the presence of other associated findings can aid in differentiating among the different causes of B-lines [58]. 

Pitfalls Associated With POCUS Examination of Kidneys

**Figure 1:**
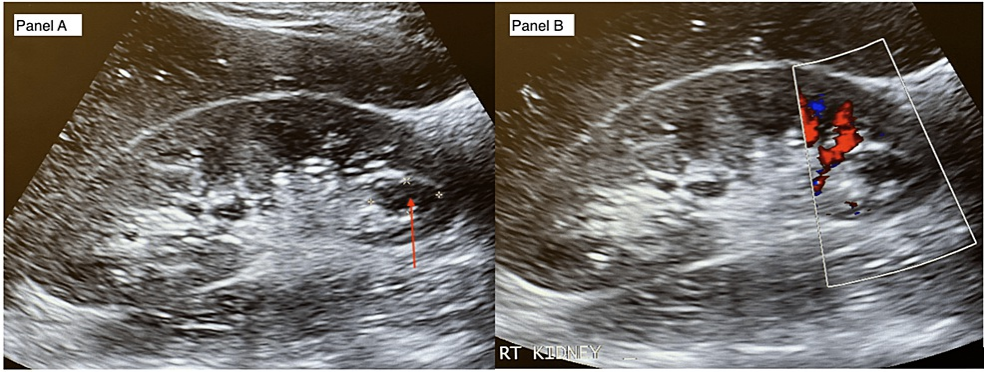
Renal cyst in the lower pole of the right kidney (red arrow) that might look like a dilated calyx (Panel A). It does not show a color Doppler effect compared to the rest of the renal pelvis (Panel B).

A common reason to perform kidney POCUS in internal medicine is to check for hydronephrosis as an indicator of the post-renal cause of acute kidney injury. Hydronephrosis can readily be identified by POCUS, but many conditions can mimic hydronephrosis, including pregnancy, dilated renal parenchymal vessels (especially in well-hydrated patients), and renal cysts [59-61]. Renal cysts are also anechoic but are well-demarcated, do not communicate with the collecting system, and do not show the Doppler effect [62] (Figure 1).

Although rare, the absence of hydronephrosis on POCUS does not entirely rule out obstruction since nondilated obstructive uropathy can occur [63]. Also, the examiner needs to be aware of the normal anatomical variations of kidney shape, like the horseshoe kidney and reduplicated renal collecting systems, that they may occasionally encounter. Hydronephrosis can be a surrogate finding in patients with renal stones since not all stones can be seen by ultrasound, but the absence of hydronephrosis does not necessarily mean the absence of stones [64]. Whenever in doubt, the examiner should consider an official ultrasound exam or other radiological modalities to look for such pathologies.

Pitfalls Associated With POCUS Examination of the Lower Extremity Venous System for Deep Venous Thrombosis 

The Society of Radiologists in Ultrasound recommends using an extended compression method (from thigh to knee) rather than the 2-region compression method (femoral and popliteal regions only) to reduce the chance of missing isolated femoral vein deep venous thrombosis (DVT) [65]. Detecting DVT using POCUS relies on adequate and careful compression during the exam and not on visualization of the clot inside the vein. An acute clot can be anechoic (black) like the flowing blood surrounding it and thus will not be seen. Some hyperechoic structures can be due to increased gain (Video 7) or very slow blood flow in the area (rouleaux formation or sludge sign) [66].

**Video 7:** Femoral vein exam showing full compressibility, excluding DVT Inside the femoral vein, an isoechoic (grey) shadow can be seen inside the anechoic (black) vessel that could be mistaken for a clot, but the vessel is fully compressible, excluding DVT. DVT: Deep venous thrombosis

Inadequate pressure during the exam may give the false impression of a positive study. To avoid this in obese patients, examiners should consider placing their hands behind the patient’s thigh and pushing anteriorly towards the probe (2-hand technique). The compression method has shown low sensitivity in detecting isolated calf vein thrombosis compared to the more proximal femoral vein area [67]. This is possibly because it is technically more difficult to obtain good images of the deep popliteal vessels compared to the more superficial and easily obtained femoral vessel images. It is also important to differentiate inguinal lymph nodes from thrombus inside a deep vein [68]. To the novice POCUS user, femoral arterial calcifications may mimic a clot if they're not able to differentiate the femoral artery from the femoral vein based on their echogenic characteristics. Less commonly, a false-negative test might be due to duplicated superficial femoral veins where the other femoral vessel with potential DVT was not examined [69,70].

Pitfalls Associated with POCUS Examination for Abdominal Aortic Aneurysm Screening

One study suggested that lack of experience and body habitus are important factors associated with difficulty visualizing and measuring the aorta. Most abdominal aortic aneurysms (AAA) are fusiform, but saccular aneurysms do exist and require follow-up as well [71,72]. They can be missed if the examination is not done carefully and systematically. A good view and measurements can be difficult when bowel gas intervenes between the aorta and the probe. In these circumstances, patience, asking the patient to bend their knees in order to relax abdominal muscles, and slow attempts to push the bowel aside using the probe can result in better image acquisition. Measurement errors and confusing the aorta with the IVC or large para-aortic lymph nodes can also occur (see Video 8) [73]. Consider other imaging modalities or sending the patient for an official ultrasound screening exam if the image is not satisfactory instead of documenting it as a negative screening.

**Video 8:** Cross-sectional view of the aorta during abdominal aortic aneurysm screening Novice POCUS users may confuse the aorta (pulsating, thicker walls) for the IVC (non-pulsating, thinner walls). POCUS: Point-of-care ultrasound, IVC: Inferior vena cava

How novice learners can improve their POCUS skills and minimize pitfalls early during POCUS training 

Table 1 summarizes what we discussed above regarding some of the common POCUS pitfalls in clinical practice relevant to internal medicine practice. 

**Table 1:** Summary of common pitfalls associated with specific POCUS exams applicable to internal medicine POCUS: Point-of-care ultrasound, IVC: Inferior vena cava, DVT: Deep venous thrombosis

POCUS exam	Pitfall	Comments
Evaluation of left ventricular contractility	Poor apical view image	Examine the patient in the left lateral decubitus position
	Not using the papillary muscle level during the left parasternal short axis view can over or underestimate contractility	Use papillary muscle level as the most optimal level to estimate contractility during the short-axis view exam
	Using only mitral valve early (E) point septal separation in patients with valvular disease	Listen for murmurs during the physical exam. Use more than one concept and view to assess left ventricular contractility.
	Left ventricular foreshortening (apex in the apical view appears rounded instead of the normal pear shape)	Slowly rotate and/or tilt the probe to 'open up' the left ventricular cavity during the exam
Evaluation of pericardial effusion	Confusing epicardial fat for pericardial effusion	Epicardial fat is more heterogeneous and moves with heartbeats. Pericardial effusion is anechoic, does not move with heartbeats, and can be seen in other cardiac views as well.
	Confusing pericardial effusion for left pleural effusion during the left parasternal long axis view	Look at the descending aorta: pericardial effusion tracks anteriorly; pleural effusion tracks posteriorly
Evaluation of right ventricular strain	Assuming RV strain changes are due to pulmonary embolism only	While some features (e.g., free right ventricular wall without hypertrophies and hypokinetics) can suggest acute right ventricular strain more, but these are not very reliable. Interpret in the appropriate clinical setting using other clinical data (e.g., history, prior echocardiography results, etc.).
Evaluation of IVC	Mistaking the aorta for the IVC	Aorta runs more posteriorly, has thicker walls, and pulsates. Inferior vena cava can be obtained from the aorta window by tilting (fanning) the probe slowly towards the patient's right side.
	The 'cylinder effect'	Slowly tilt (fan) the probe right and left over the full diameter of the IVC and measure the maximal diameter
Evaluation of the lung	Not examining enough lung areas and premature closure of findings	Look at multiple lung windows (similar to auscultation)
	Not appreciating lung sliding and attributing that to pneumothorax only	Interpret findings according to clinical data. Know the differential diagnosis of absent pleural sliding. Look for lung point during the exam.
	Attributing B-lines to cardiogenic pulmonary edema only	Know the differential diagnoses of pulmonary B-lines and the pathologies associated with them; know how to differentiate between them based on ultrasound features and clinical data.
Evaluation of the kidneys	Confusing hydronephrosis with renal cysts or dilated parenchymal vessels	Cysts are usually well-rounded, well-demarcated, do not communicate with the renal collecting system, and do not show the Doppler effect. Examine the kidneys from pole to pole in both longitudinal and cross-sectional views.
	Ruling out kidney stones when POCUS does not show them or does not show hydronephrosis	Computed tomography scan is more sensitive than ultrasound for detecting renal stones.
	Confusing pathology with normal anatomical variants	Learn the common anatomical variations of the kidneys and collecting system.
Evaluation for lower extremity DVT	Inadequate pressure	Without causing discomfort to the patient, compress until the artery also starts to slightly collapse. Consider placing one hand behind the patient’s thigh and pushing anteriorly towards the probe (2-hand technique).
	Mistaking inguinal lymph nodes for clots inside the femoral vein	Scan the proximal and distal structures. Lymph nodes are self-contained compared to veins, which can still be visualized with their attributes.
	Mistaking arterial calcifications for clots	Femoral artery has thicker walls and pulsates, and is lateral to the femoral vein, which is thinner, does not pulsate, and is easier to compress if no clot is present. Calcifications are more hyperechoic than clots and adhere to the walls of the artery.
Evaluation of the abdominal aorta for an aneurysm	Not able to visualize the aorta due to bowel gas	Ask the patient to bend their knees in order to relax their abdominal muscles. Use slow probe pushes to attempt to move the bowel aside
	Missing saccular aneurysm	Be systematic. Learn how a saccular aneurysm looks with ultrasound compared to a fusiform aneurysm.
	Confusing IVC for the aorta	The aorta lies to the left of the IVC, has thicker walls, and pulsates.

Handheld devices are now more available than ever and are more affordable than traditional cart-based ultrasound machines. Interested physicians or programs should try to obtain one or more of the handheld ultrasound devices to improve their POCUS skills. The portability of handheld devices can encourage physicians to practice POCUS more because it is of immediate availability to them compared to cart-based ones. Novice learners can start by using the larger machines first if they appreciate images and anatomy being clearer with the larger machines.

Image acquisition is usually more difficult than image interpretation. Ultrasound technicians, with their immense practical experience, can be a valuable source for physicians starting to use POCUS to learn maneuvers and different probe movements that help find the best window for each exam. When a consultative ultrasound exam is done for a patient, the treating physician can compare their findings to the official report, and if there is any discrepancy, they can repeat their POCUS exam again or seek help from an expert as feedback to improve their skill for the next exam.

Deliberate practice, with focus on and repetition of a particular aspect of skill or exam (for example, obtaining a 4-chamber cardiac view) and receipt of direct feedback from an expert during these practices, has shown to improve procedural and psychomotor skills. Although it has not been extensively studied for sonography skills, it is a promising method to improve POCUS skills. It can be part of the POCUS curriculum so that learners can practice particular POCUS skills that seem to be more difficult for them to learn compared to others [74-76]. As stated before, this can be done with the help of a software platform where POCUS images can be saved and reviewed by experts at their convenience. 

Interested physicians can pursue any of the certificates of completion available at this time, with the expectation that more will be available in the future [77-79]. Such certificates can serve as a good way towards proficiency, although they do not grant proficiency themselves. They can enhance a user’s basic POCUS knowledge with online modules and testing and, to some extent, image acquisition through hands-on courses and reviews by a panel of POCUS experts. It is not yet clear whether certification and re-certification will be a requirement in the future for physicians before they can implement POCUS in their practice. 

## Conclusions

The utilization of POCUS is anticipated to undergo further expansion, whereby a greater number of internal medicine trainees and practitioners are expected to incorporate it into their medical practice across diverse clinical environments in the imminent future. Nonetheless, similar to any other clinical tool, it is imperative for novice users to possess a comprehensive understanding of its limitations and remain cognizant of the potential pitfalls associated with its implementation, in addition to being acquainted with its merits and advantages. Such awareness is crucial to preventing potential harm to patients, mitigating the risk of erroneous decision-making, and averting unnecessary delays in conducting appropriate investigations and administering suitable management strategies. Moreover, this knowledge can enable practitioners to maximize the potential benefits of investing considerable time, effort, and financial resources in mastering the utilization of POCUS. With more use, more studies and research can be conducted to inform best practices and add to the body of evidence related to POCUS applications in internal medicine.
